# Perineal approach for surgical treatment in a patient with retro-rectal tumor: a case report and review of the literature

**DOI:** 10.1186/s13104-015-1457-5

**Published:** 2015-09-24

**Authors:** Mohamed Tarchouli, Aziz Zentar, Moulay Brahim Ratbi, Abdelhak Bensal, Mohamed Reda Khmamouche, Abdelmounaim Ait Ali, Ahmed bounaim, Mohammed Elfahssi, Khalid Sair

**Affiliations:** Department of Digestive Surgery, Faculty of Medicine and Pharmacy, Mohammed V Military Hospital, Mohammed V University, Rabat, Morocco; Department of Oncology, Faculty of Medicine and Pharmacy, Mohammed V Military Hospital, Mohammed V University, Rabat, Morocco

**Keywords:** Retro-rectal tumor, Surgical management, Perineal approach

## Abstract

**Background:**

Retrorectal tumors in adults are very rare and little known condition. These tumors, often misdiagnosed or mistreated, should be completely excised because of the potential for malignancy or infection. A suitable operative approach is the key to the successful surgical management.

**Case presentation:**

We report the case of a 45-year-old Arab male who presented with chronic pelvic pain accompanied by straining to defecate and dysuria. The clinical examination showed a painless mass in the left perineal area. Pelvic magnetic resonance imaging and computed tomography scan demonstrated a huge and well-limited pelvic mass causing displacement and compression of the rectum and bladder. Although the large size of the mass (>7 cm in the greater diameter), it was successfully and completely excised through only perineal approach without undertaking coccygectomy or sacrectomy. The histopathological study revealed a low-grade leiomyosarcoma. The patient is currently in 4-years follow-up with no signs of recurrence or metastasis.

**Conclusion:**

Even large retro-rectal tumors may be successfully excised by the perineal approach especially in carefully selected patients, but require extensive knowledge of pelvic anatomy and expertise in pelvic surgery.

## Background

Retro-rectal tumors are a very rare entity in the adult population with an incidence of about 1 in 40,000 patients [1]. Being mostly congenital, they develop in the retro-rectal space and range from benign cysts to complex malignant masses. Due to their non-specific presentation, these lesions are often misdiagnosed and may remain undetected for a long time. Because of delayed diagnosis, the tumors can grow to a large size and may invade the surrounding vascular and neurological structures making the management more difficult and complicated. Surgical resection is the best therapeutic option but may be challenging, especially in determining the most appropriate approach.

With this case report we attempt to describe the main features related to this disease and highlight key points to improve its safe and successful surgical management particularly with perineal approach.

## Case presentation

A 45-year-old Arab male presented with a 4-years history of chronic pelvic pain accompanied by straining to defecate and dysuria, but without perineal discharge, rectal bleeding, headaches or weight loss. The patient reported to be operated for anal fistula 10 years ago with satisfactory clinical evolution. His family history was unremarkable and no smoking or chronic alcoholism were noticed. In the proctologic (knee-chest) position, physical examination revealed a 4–5 cm, soft and painless mass in the left perineal area. This mass seemed freely mobile with lower edge extending to the pectinate line but the upper limit was not perceived. No inflammatory signs, fistulous tracts or postanal skin dimple were observed. The mucosa was normal on digital rectal examination and exterior pressure was detected in the posterior region.

**Fig. 1:**
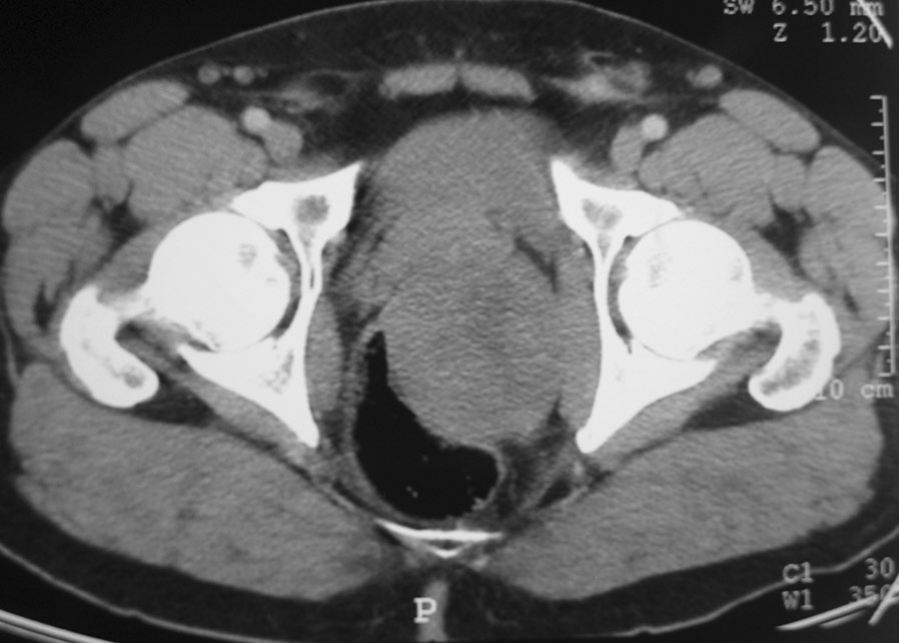
Computed tomography scan findings: axial section demonstrating a bulky and well-circumscribed mass developing in the pelvis with evident displacement of the rectum and bladder

**Fig. 2:**
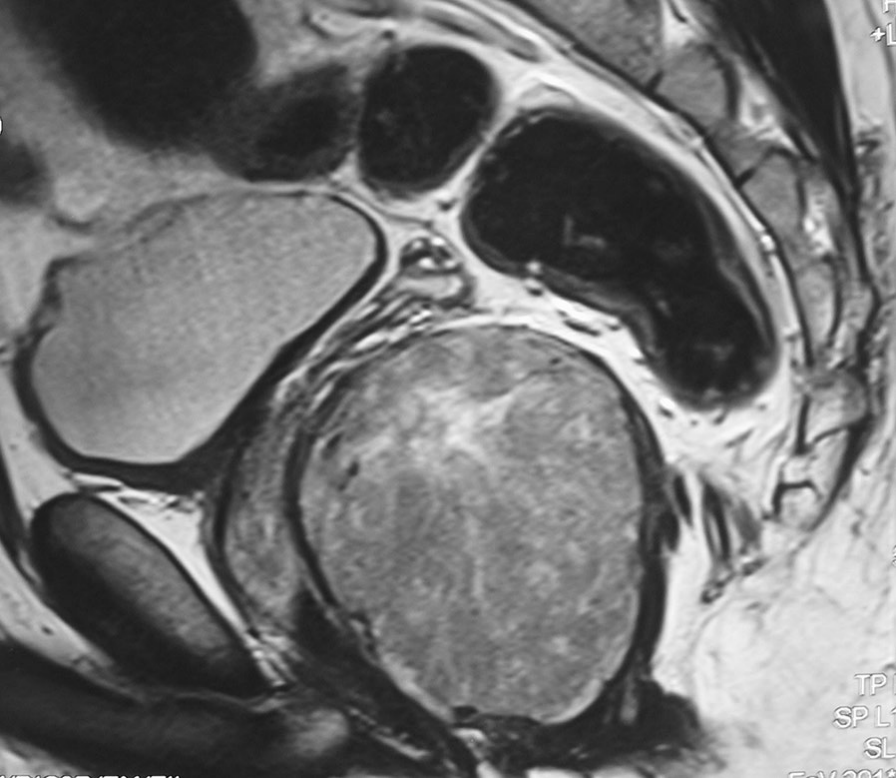
Magnetic resonance imaging findings: sagittal T2-weighted image showing a large pelvic mass with high signal intensity and heterogeneous content but without bone or bladder invasion or any evident communication with the lumen of the rectum

Abdominal ultrasound detected a hypoechoic solid pelvic mass with regular contours and heterogeneous echostructure, but relationship with the surrounding structures was not clear. On computed tomography (CT) scan the lesion appeared as a large heterogeneous mass measuring approximately 7 cm in diameter. This mass, developed in the latero-rectal space, extended to the left side of the pelvis causing displacement and compression of the rectum and urinary bladder. There was no pelvic effusion, deep lymph nodes or bone destruction (Fig. 1). Additionally, pelvic magnetic resonance imaging (MRI) confirmed the presence of a 7 cm × 7 cm well-limited lesion in continuity with the left rectal wall and extending from the fourth sacral vertebra (S4) level to the coccygeal region. The lesion had low signal intensity on T1-weighted images and heterogeneous high signal intensity on T2-weighted images without any signs of bladder invasion or any evident communication with the lumen of the rectum **(**Fig. 2**)**. Moreover, rectoscopy detected a bulging mass with intact mucosa in the posterior wall of the rectum. Routine blood tests and serum tumor markers including carcinoembryonic antigen, cancer antigen 19–9, cancer antigen 125, prostatic specific antigen and α-fetoprotein were within normal range.

**Fig. 3:**
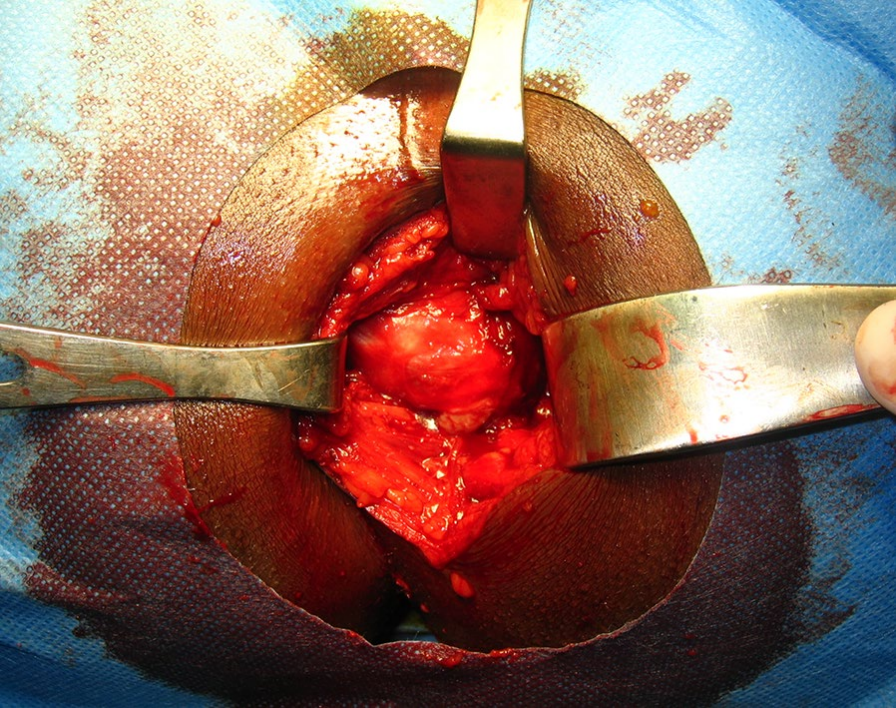
Operative view

**Fig. 4:**
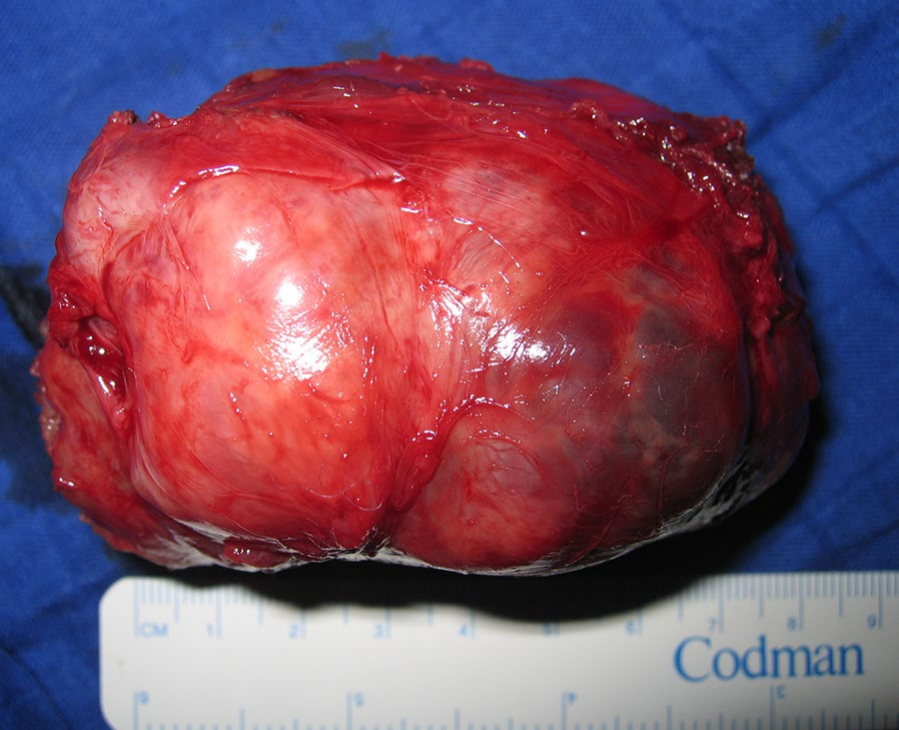
Resected specimen after complete surgical removal

According to these findings, the patient was planned for surgical excision without a preoperative fine-needle biopsy. We chose a perineal approach under spinal anesthesia. The patient was placed in the lithotomy position. Through a vertical para-anal skin incision of about 10 cm centered on the lesion, the subcutaneous planes were divided and the retrorectal space was exposed. We discovered a soft tumor-mass, deeply extending into the ischiorectal fossa, and intimately adherent to the posterior face of the rectum and levator ani, but with a cleavage line making easy the tumor dissection from the surrounding tissue. The mass was carefully dissected (essentially blunt dissection) and completely excised, with special attention to avoiding injuries to the sphincter complex and rectal perforation **(**Figs. 3, 4). After tumor excision, the wound was closed in anatomical planes and drains left in the retro-rectal space for 48 h.

Pathological examination of the resected specimen revealed an encapsulated mass measuring 82 × 70 × 60 mm. Microscopically, the tumor was composed of spindle shaped cells arranged in interlacing fascicles with focal myxoid change and areas of necrosis. Number of mitoses was 4 per 10 high power fields (HPF) and all surgical margins were free of disease. In addition, tumor cells exhibited positive staining for smooth muscle actin and desmin, and negative staining for CD-117, CD-34 and S-100 protein. With these findings, final diagnosis was low-grade leiomyosarcoma. According to the low grade of the tumor and the complete surgical excision, no adjuvant treatment was decided and the patient was discharged after an uneventful postoperative period. He is currently in 4-years follow-up with no evidence of recurrence or metastasis. He underwent periodically physical examination with abdomen/pelvic CT every 3 months for 2 years, then every 6 months for the next 2 years.

## Discussion

The retro-rectal or pre-sacral space is the virtual anatomic area limited anteriorly by the fascia propria of the rectum, posteriorly by the presacral fascia, superiorly by the peritoneal reflection, and inferiorly by the recto-sacral fascia (Waldeyer’s fascia) and the supralevator space. The lateral boundaries are the lateral stalks of the rectum, the ureters and the iliac vessels [2, 3]. This space contains multiple embryologic structures derived from various tissues; consequently a heterogeneous group of both benign and malignant tumors can occur in this area. Although infrequent in the adult population, tumors of the retro-rectal space include different types of lesions and may be congenital or acquired. Traditionally, these tumors are classified according to the histologic origin and can be divided into congenital, inflammatory, neurogenic, osseous and miscellaneous types (Table 1) [2, 4]. Congenital tumors are the most frequent type, and solid lesions are more likely to be associated with malignancy than are cystic lesions [5].

**Table 1:** Classification of retro-rectal tumors

*Congenital* (55–65 %) Developmental cyst (epidermoid, dermoid) Cystic hamartoma (tailgut) Teratoma Rectal duplication Adrenal rest tumor Anterior sacral meningocele Chordoma Teratocarcinoma *Neurogenic* (10–12 %) Neurofibroma Ependymoma Neurilemoma (Schwannoma) Ganglioneuroma Neuroblastoma Neurofibrosarcoma *Inflammatory* (5 %) Granulomas Perineal or pelvirectal abscess Diverticulitis Crohn’s disease	*Osseous* (5–11 %) Osteoma Simple bone cyst Ewing’s tumor Giant cell tumor Chondrosarcoma Osteosarcoma *Miscellaneous* (12–16 %) Metastatic disease Lipoma/liposarcoma Fibroma/Fibrosarcoma Lymphangioma Carcinoid Desmoid tumor Leiomyoma/leiomyosarcoma Endothelioma Pelvic ectopic kidney

Retro-rectal tumors, typically slow growing, may be completely asymptomatic over prolonged periods of time. Consequently, they are often only discovered incidentally during examination for unrelated physical complaints. The symptoms are usually nonspecific and attributed to compression or invasion of surrounding structures, as was observed in our patient. Symptomatic patients may present vague pain in the perineal area, chronic constipation, rectal or urinary incontinence or sexual dysfunction. Some tumors can lead to perineal discharge with midline dimpling in the sacrococcygeal area, and may be confused with perianal suppurations and complicated fistulas [5, 6]. Nevertheless, most of these lesions are easily detectable on digital rectal examination.

Although pelvic plain films can reveal indirect signs such as the bone destruction typical of malignancy and the presence of calcifications in mature teratomas, diagnosis requires modern imaging techniques for preoperative evaluation of retro-rectal tumors. Providing excellent morphological description of these tumors, CT and MRI have become the best diagnostic modalities to date. CT scan is used to distinguish whether tumors are cystic, solid or mixed and to assess for sacral involvement or invasion to pelvic structures. Whilst MRI, with his higher resolution in soft tissue, is particularly useful in delineating soft-tissue planes and precisely evaluating the relationships of the mass with bones, muscles and nerves [7]. These parameters are extremely important in determining level and extent of resection and deciding the most appropriate surgical approach. On the basis of such considerations, we decided to submit our patient to pelvic CT scan in conjunction with pelvic MRI, which gave us precise information proved crucial for our preoperative planning. Abdominal ultrasound was initially performed only as routine imaging technique for pelvic pain. Otherwise, the role of preoperative biopsy is controversial. However most authors believe that it should be avoided because of the risk of bleeding, tumor seeding along the biopsy tract, infection of cystic lesions and lethal meningitis in the case of meningocele [6, 8]. For these reasons, the biopsy was not performed in our patient.

In appropriate surgical candidates, complete surgical excision with clear resection margins remains the cornerstone option for surgical management of retro-rectal tumors, even if asymptomatic and apparently benign. The possibility of malignancy and the risk of eventual dystocia in women of childbearing age, of degeneration in teratomas or infection in cystic tumors have been reported as the reasons for necessity of operative extirpation [5]. Definitely, a suitable approach is the key to the successful management of these tumors [9]. Thus, the choice of surgical approach, based on preoperative imaging, depends on the size, location, and spatial relationship of the lesion with adjacent structures. An important consideration is the likelihood of malignant disease, which mandates a more aggressive surgery.

Generally, three different surgical approaches are commonly used in the resection of retrorectal tumors: anterior, posterior, and combined approaches. The anterior or abdominal approach is recommended for larger (more than 5 cm) and highlying (the lower margin of the lesion is above the level of S4) retro-rectal tumors, because it allows an excellent exposure of pelvic structures, bleeding control, and easier mobilization of rectum [3, 10]. Although experience with minimally invasive techniques is still limited, laparoscopic approach for some cases of benign retro-rectal tumors has been recently reported [11, 12]. In contrast, small sized (less than 5 cm) and low-lying tumors (the upper limit of the lesion is below the level of S4) can be removed by the posterior approach [2, 13]. This procedure is preferred by most surgeons, especially when proximal extent of the mass is accessible on digital rectal examination, because it is faster and easier to do. Either a transperineal or parasacral route may be used. Furthermore, the combined abdominoperineal approach is required in excessively large tumors extending both proximally and distally to the S4 or apparently malignant disease involving adjacent organs without chance of surgical removal by the only abdominal or perineal approach.

Abdominoperineal approach seemed to be the most appropriate procedure in our patient, because the tumor extended below the level of S4 and its upper limit was not reachable on digital rectal exploration. However, despite the large size of the mass and its intrapelvic extension, considered as not suitable for the only perineal approach, we chose it to avoid laparotomy and its potential complications. We obtained optimal exposure with good access to both the tumor and the retrorectal space through a longitudinal para-anal incision centered on the lesion without the necessity of abdominal access. We believe that the perineal approach was particularly feasible in our patient because of the absence of rectal and sacral invasion. This enabled us to achieve an easy dissection of the intrapelvic component of the tumor using retrorectal fat tissue as a cleavage line between tumor and rectum.

Identification and transaction of the anococcygeal ligament facilitates exposure of the mass and dissection of the plane between the lesion and the mesorectum. In addition, many authors advocate routine coccygectomy in order to obtain sufficient exposure allowing the complete excision of the mass. Teratomas and all cystic lesions are known to often arise from the coccyx, thus “en bloc” coccygeal resection with these tumors seems necessary for lower rate of recurrence. Currently, this attitude is not recommended unless the coccyx is frankly invaded by a malignant or highly suspected malignant lesion [8]. If sacrectomy is needed, particularly in the case of sacral invasion, it should preserve at least one side of the second sacral vertebra (S2) in order to avoid urinary and rectal sphincter incontinence. Tumors associated with extensive involvement of the rectal wall require proctectomy and are typically best managed using a combined abdominoperineal approach. The perineal approach provide good access to the caudal component, but the major drawbacks are the absence of control over pelvic vessels and the potential for injury to the lateral pelvic nerves [5, 8]. In our patient, the tumor was completely excised without performing coccygectomy or sacrectomy but with a great care to avoid injury to the sphincter complex or rectal wall. Although the tumor described in our case was larger than 5 cm, it was successfully and completely resected through only perineal approach. Regarding to the complete surgical excision and the absence of recurrence after 4 years’ follow-up, we believe that our attitude was correct and we think that even large retro-rectal tumors may be safely excised by the perineal approach especially in carefully selected patients, such as low-lying and apparently benign lesions.

Finally, it is important to emphasize that the successful management of retro-rectal tumors requires extensive knowledge of pelvic anatomy and expertise in pelvic surgery. Consequently, optimal treatment can be achieved only by a multidisciplinary team involving colorectal surgeons, neurosurgeons, orthopedic and plastic surgeons especially with tumors involving the sacrum and sacral nerve roots.

## Conclusion

Retro-rectal tumors are an uncommon disease with often nonspecific clinical presentation which makes diagnosis late and difficult. Therefore, diagnosis of these lesions requires a high index of suspicion, and should be considered in any patient with palpable mass on rectal examination. Complete surgical resection with negative margins is the best treatment even in asymptomatic patients. The perineal approach is feasible, minimally invasive and safe option, providing complete recovery for carefully selected patients with low-lying retro-rectal tumors. This approach should be included in the therapeutic arsenal of colorectal surgery. Finally, a multidisciplinary team is likely to improve the rate of successful treatment of these specific lesions.

## Consent

Written informed consent was obtained from the patient for publication of this Case Report and accompanying images. A copy of the written consent is available for review by the Editor-in-Chief of this journal.

## Abbreviations

CT: computed tomography
MRI: magnetic resonance imaging
S4: fourth sacral vertebra
S2: second sacral vertebra
HPF: high power field
